# Inability to Utilize Retrograde Cardioplegia due to a Persistent Left Superior Vena Cava

**DOI:** 10.1155/2017/4671856

**Published:** 2017-12-03

**Authors:** Rohesh J. Fernando, Sean D. Johnson

**Affiliations:** Cardiothoracic Section, Department of Anesthesiology, Wake Forest School of Medicine, Medical Center Boulevard, Winston Salem, NC 27157-1009, USA

## Abstract

A persistent left superior vena cava is a congenital abnormality that affects a minority of the general population. While this finding is not hemodynamically significant in all patients, failure to recognize the altered anatomy in any of these patients can be consequential during procedures such as central venous catheter placement, pacemaker/defibrillator wire placement, and use of retrograde cardioplegia during cardiac surgery. We present a case of an intraoperative diagnosis of a persistent left superior vena cava that altered the original plan to arrest the heart using retrograde cardioplegia. Echocardiography was instrumental in this diagnosis and avoided potentially inadequate myocardial protection during cardiopulmonary bypass.

## 1. Introduction

Despite being the most common vascular anomaly of the thoracic venous system, a persistent left superior vena cava (PLSVC) remains an uncommon finding in the general population [1]. It results when the left superior cardinal vein fails to regress. The incidence is estimated to be 0.1%–0.3% within the general population, although a higher prevalence has been described in patients with congenital heart disease (CHD). In these CHD patients, the prevalence has been reported to be as high as 5% when using echocardiography and 11% with invasive angiography [2]. Variations exist such that the innominate vein and/or right superior vena cava (SVC) may or may not be present. Generally, the PLSVC communicates with the right atrium via the coronary sinus. Less commonly, the PLSVC can drain into the left atrium, thereby causing a right to left shunt. Other associated abnormalities include bicuspid valve, coarctation of the aorta, cor triatriatum, and cardiac septal defects [3]. In 80%–90% of patients with a PLSVC, however, it is of no hemodynamic consequence [1]. We present a case of a PLSVC that was incidentally discovered during cardiac surgery. Written informed patient consent was obtained to publish this case report.

## 2. Case Presentation

**Figure 1:**
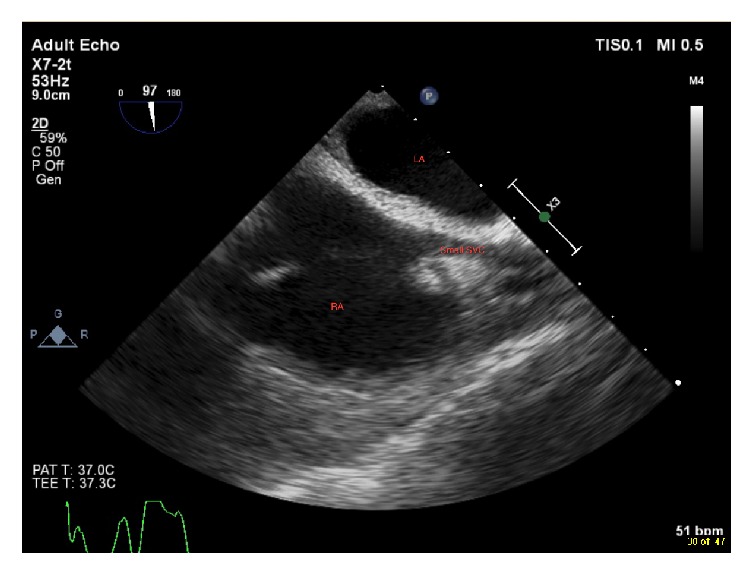
Midesophageal bicaval view showing small superior vena cava (SVC). RA = right atrium; LA = left atrium.

A 46-year-old male with a history significant for atrial fibrillation, dilated aortic root, and bicuspid aortic valve with severe aortic regurgitation (AR) presented to the operating room for elective aortic valve replacement, aortic root replacement, and Maze procedure. His preoperative transthoracic echocardiogram (TTE) was notable for normal biventricular function, severe AR, and a possible patent foramen ovale (PFO). No details about the coronary sinus were provided on the TTE report. Intraoperatively, the patient underwent an uneventful induction of general anesthesia. Transesophageal echocardiography (TEE) was then performed and confirmed the preoperative findings with the exception that there was no PFO identified. Other notable findings, however, were also discovered on TEE. During placement of a central venous catheter in the right internal jugular vein, TEE was used to visualize the guidewire. It was noted at this time that the right SVC appeared small (Figure 1).

**Figure 2:**
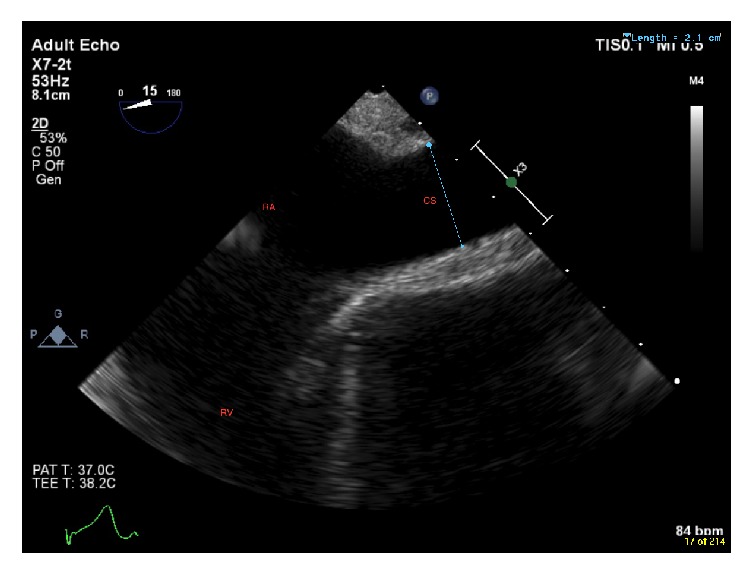
Deep midesophageal 4-chamber view showing a dilated coronary sinus (2.1 cm). RA = right atrium, RV = right ventricle, and CS = coronary sinus.

**Figure 3:**
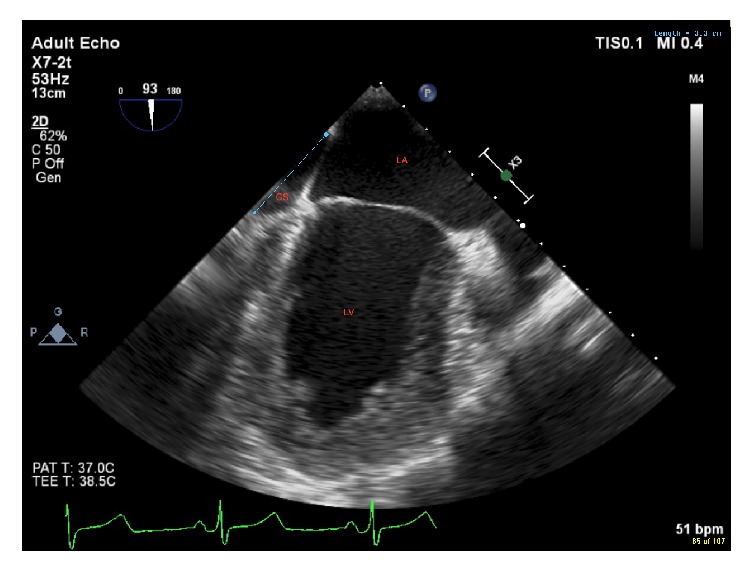
Midesophageal 2-chamber view showing dilated coronary sinus (3.3 cm). LA = left atrium, LV = left ventricle, and CS = coronary sinus.

In addition, the coronary sinus was observed to be significantly dilated, measuring 2.1 cm in the deep midesophageal 4-chamber view (Figure 2) and 3.3 cm in the midesophageal 2-chamber view (Figure 3).

These echocardiographic findings raised suspicion for a PLSVC. A left-sided intravenous line was in place so a bubble study was attempted. Injection of agitated saline showed bubbles appearing in the coronary sinus prior to entering the right atrium (Video 1 in Supplementary Material), although the study was limited by the relatively low number of bubbles. The suspicion for a PLSVC was communicated to the surgical team given the possible implications for retrograde cardioplegia administration. However, at that time, they were not able to visually identify a PLSVC.

The patient was cannulated for CPB. Given the severe aortic insufficiency, retrograde was preferred over antegrade cardioplegia. Once CPB was established, retrograde cardioplegia administration was attempted but there was difficulty achieving pressurization of the coronary sinus. Since the patient was on CPB by this time, a more thorough visual examination of the coronary sinus was possible without hemodynamic instability. Further inspection by the surgical team confirmed that there indeed was a PLSVC in continuity with the coronary sinus and antegrade cardioplegia was used to arrest the heart while carefully monitoring the left ventricle for distention using TEE. The heart was successfully arrested and the patient underwent MAZE along with aortic valve and root replacement. The patient was weaned from CPB uneventfully and was taken to the intensive care unit postoperatively. No intervention was performed for the PLSVC.

## 3. Discussion

Recognizing a PLSVC is most important during central venous cannulation, pacemaker and/or defibrillator placement, and cardiac surgery with use of retrograde cardioplegia [4]. Cases have been described where central venous catheters placed through the left internal jugular vein for vasopressor support were unintentionally placed into the tract continuous with the coronary sinus [5, 6]. Particularly when the left subclavian vein is used for access, as a result of the abnormal proximity to the coronary sinus, there is additional concern for the catheter and associated guidewire causing arrhythmias, hemodynamic stability, and perforation of the heart [7, 8]. Some authors have used catheters that were placed in the coronary sinus for drug and volume administration, although close proximity of the tip of the catheter to the coronary sinus could have the same risks as the guidewire [9]. In addition, pulmonary artery catheter and pacemaker lead placement can be difficult due to a tortuous anatomy [7, 10].

A PLSVC can be confirmed by many radiologic techniques, including angiography, computed tomography, and magnetic resonance imaging [11]. Diagnosis by echocardiography is primarily based on a dilated coronary sinus (normal diameter is less than 1 cm). In addition, the diagnosis can be supported by injection of contrast through a left-sided intravenous line with demonstration of contrast appearing in the coronary sinus prior to the right atrium [12]. Failure to diagnose a PLSVC and use of retrograde cardioplegia alone can result in inadequate myocardial protection and cardiogenic shock. Diagnosis should also be followed by an evaluation for other congenital abnormalities such as atrial and/or ventricular septal defects, cor triatriatum, coarctation of the aorta, and endocardial cushion defects [7, 13, 14]. Regardless of the presence of any associated abnormalities, it is imperative to promptly communicate the presence of a PLSVC to the surgical and perfusion team.

In conclusion, we present a case where a patient with a previously undiagnosed PLSVC presented for cardiac surgery. The preoperative TEE exam raised suspicion for a PLSVC and was instrumental in the ultimate decision to use antegrade cardioplegia to arrest the heart on CPB. Retrograde cardioplegia cannot be relied upon with this vascular anomaly due to the potential for poor myocardial protection. Although a PLSVC is uncommon within the general population, this case demonstrates echocardiographic findings that must be readily recognized by perioperative echocardiographers.

## Supplementary Materials

Supplementary Materials Video 1. Deep midesophageal 4-chamber view from a transesophageal echocardiogram showing agitated saline bubbles first entering the coronary sinus and subsequently moving into the right atrium.
